# Long-Term Treatment of Azathioprine in Rats Induces Vessel Mineralization

**DOI:** 10.3390/biomedicines9030327

**Published:** 2021-03-23

**Authors:** Mirjam Schuchardt, Jaqueline Herrmann, Cornelia Henkel, Milen Babic, Markus van der Giet, Markus Tölle

**Affiliations:** 1 Department of Nephrology and Medical Intensive Care, Charité–Universitätsmedizin Berlin, 12203 Berlin, Germany; mirjam.schuchardt@charite.de (M.S.); Jaqueline.Herrmann@charite.de (J.H.); Cornelia.Henkel@charite.de (C.H.); Milen.Babic@charite.de (M.B.); Markus.Toelle@charite.de (M.T.); 2 Department of Biochemistry, Chemistry and Pharmacy, Freie Universität Berlin, 14195 Berlin, Germany

**Keywords:** aging, azathioprine, calcification, mineralization, senescence-associated secretory phenotype

## Abstract

Medial vascular calcification (mVC) is closely related to cardiovascular disease, especially in patients suffering from chronic kidney disease (CKD). Even after successful kidney transplantation, cardiovascular mortality remains increased. There is evidence that immunosuppressive drugs might influence pathophysiological mechanisms in the vessel wall. Previously, we have shown in vitro that mVC is induced in vascular smooth muscle cells (VSMCs) upon treatment with azathioprine (AZA). This effect was confirmed in the current study in an in vivo rat model treated with AZA for 24 weeks. The calcium content increased in the aortic tissue upon AZA treatment. The pathophysiologic mechanisms involve AZA catabolism to 6-thiouracil via xanthine oxidase (XO) with subsequent induction of oxidative stress. Proinflammatory cytokines, such as interleukin (IL)-1ß and IL-6, increase upon AZA treatment, both systemically and in the aortic tissue. Further, VSMCs show an increased expression of core-binding factor α-1, alkaline phosphatase and osteopontin. As the AZA effect could be decreased in NLRP3^−/−^ aortic rings in an ex vivo experiment, the signaling pathway might be, at least in part, dependent on the NLRP3 inflammasome. Although human studies are necessary to confirm the harmful effects of AZA on vascular stiffening, these results provide further evidence of induction of VSMC calcification under AZA treatment and its effects on vessel structure.

## 1. Introduction

Vascular disease in general and arteriosclerosis in particular remain a major cause of cardiovascular morbidity and mortality, not only in patients suffering from chronic renal failure, but also in elderly patients. Up to now, convincing clinical therapy concepts are not available [1]. A longitudinal study with kidney transplant patients revealed substantial medial vascular calcification (mVC) within four years even when cardiovascular mortality decreases after transplantation [2,3]. Beside the influence of uremic toxins on vascular smooth muscle cell (VSMC) physiology [4] in patients with chronic kidney disease (CKD), evidence exists that immunosuppressive therapy can influence signaling pathways in vascular cells and thereby affect the progression of vascular alterations [5]. One of those potent immunosuppressive drugs is azathioprine (AZA) [6,7]. Even though AZA has been replaced in patients who have undergone solid organ transplantation [6], it is still routinely used as a treatment of several auto-immune diseases [7,8,9]. Several indications exist that AZA treatment has an impact on the cardiovascular risk and pathophysiology of the vessel wall [8,9]. In a previous study, we found an induction of mVC and oxidative stress upon treatment with the cleavage product of AZA, 6-mercaptopurine (6-MP), using an in vitro model with rat VSMCs [10]. Extensive research in the field of VSMC calcification revealed the involvement of several mechanisms including osteogenic, senescence and inflammatory signaling. VSMCs are characterized by a phenotype shift from a contractile to a secreting cell called the senescence-associated secretory phenotype (SASP) [11,12,13]. Recently, Shanahan’s group characterized the SASP of VSMCs by gene expression of several calcification markers, e.g., alkaline phosphatase (ALP), interleukin (IL)-1ß and p21 [13]. In the pathogenesis of CKD, inflammation and calcification are present in patients, even in early CKD stages [14]. A relevant role of NLRP3 inflammasome activation and IL-1ß production has been reported for the calcification process [15]. At least in vitro, IL-1ß stimulates VSMC calcification, while NLRP3 knockdown inhibits it [15]. Further, it has been shown that IL-6 induces calcification of human VSMCs in vitro [16]. In patients treated with AZA, the IL-6 plasma level and oxidative stress markers increase [17]. In the current study, the calcifying effect of AZA was investigated in an in vivo rat model treated with AZA for 24 weeks to support evidence of possible harmful effects on vessel stiffening in patients who underwent long-term AZA treatment over years.

## 2. Materials and Methods

### 2.1. Animals

Male Wistar rats (*n* = 29) were purchased from Janvier (Le Genest-Saint-Isle) at the age of 8 weeks. At the age of 14 weeks, rats were divided randomly into 2 groups. For 24 weeks, the treatment group (*n* = 16) received AZA (10 mg/kg body weight) orally via the drinking water and the control group (*n* = 13) received no therapy. The drinking solution for the animal treatment was prepared daily and the amount of drinking water per cage was monitored to determine the mean AZA uptake per animal and day. Health and body weight statuses were monitored in short intervals during the trial period. After 24 weeks, animals were sacrificed by intraperitoneal injection of pentobarbital (400 mg/kg body weight). Blood was obtained and organ specimens were prepared for cryofixation and formalin fixation. Heparin plasma was collected by centrifugation, aliquoted and frozen at −80 °C until use. NLRP3^+/+^ and NLPR3^−/−^ mice (C57/BL6 background) were sacrificed by intraperitoneal injection of pentobarbital (200 mg/kg body weight). Blood was obtained in heparinized tubes; heparin plasma was collected by centrifugation and aliquoted frozen at −80 °C until use. Rats and mice were kept on a 12 h light/dark cycle with chow and water ad libitum. The room temperature was around 22 °C. Chow was purchased from Ssniff (Soest).

### 2.2. Blood Parameters

Blood parameters were analyzed in heparin plasma by a blood dry chemistry analyzer (Fuji) for alkaline phosphatase (ALP), calcium (Ca) and inorganic phosphate (IP) according to the manufacture’s recommendations. Parathyroid hormone (PTH) (Rat Biointact PTH ELISA, Immutopics), serum amyloid A (SAA, Rat SAA CLIA Kit, ElabScience), 8-oxo-2-deoxyguanosine (MyBioScource), Fetuin-A (LS Bio), Caspase-1 (Biorbyt) and MGP (LS Bio) plasma concentrations were measured via ELISA according to the manufacture’s protocol. Plasma concentration of 23 cytokines was determined using Milliplex (Millipore) according to the manufacturer’s instructions.

### 2.3. Histological Staining

Histological stains of the aorta were performed in 4 µm sections of paraffin-embedded tissue. Deparaffinized sections were subsequently stained using von Kossa staining. Images were acquired using a Zeiss AxioVert 200M light microscope with ZEN2 software (Zeiss, blue edition).

### 2.4. Quantification of the Tissue Calcium Content

Extracellular calcium content of different vascular beds was determined as previously published [10,18].

### 2.5. Ex Vivo Stimulation of Aortic Tissue

The thoracic aorta of NLRP3^+/+^ and NLRP3^−/−^ mice was stimulated with calcification medium: Dulbecco’s modified Eagle medium (DMEM) 4.5 g/L glucose, supplemented with 15% fetal calf serum, 1% penicillin/streptomycin, 5 mmol/L sodium hydrogen phosphate and 0.284 mmol/L ascorbic acid. In co-stimulated samples, medium supplemented with 0.1 mmol/L AZA was used. The aortic tissue was stimulated for 14 days, and the medium was changed every three days. Afterwards, the aortic tissue was decalcified for 24 h with 0.6 mol/L hydrochloric acid (HCl). The tissue was dried, and dry weight was measured for normalization.

### 2.6. Gene Expression

Cryoconserved tissue was homogenized using the Tissue Ruptor with disposable probes (Qiagen). RNA was isolated using Trizol^®^ (Fisher Scientific, Waltham, MA, USA) and afterwards reverse transcribed using the High-Capacity cDNA Reverse Transcription Kit™ (Applied Biosystems) according to the manufacture’s protocol. Quantitative determination of mRNA expression was conducted using the iQ™ SYBR Green supermix and the CFX384 real-time PCR detection system (Biorad, CFX software version 3.1). The oligonucleotide sequences are given in Supplementary Table S1. GAPD, Ppia and Rpl13A were used as housekeeping normalization for each sample. The Ct average of the respective control mice was used for Ct normalization.

### 2.7. Statistical Analysis

Data are provided as mean ± SEM. Statistical analysis was performed using GraphPad Prism software (version 6.0). To evaluate differences between treatment groups, the Mann–Whitney U test or Wilcoxon matched pairs test was applied. A *p*-value < 0.05 was considered as statistically significant.

## 3. Results

### 3.1. In Vivo Model

The AZA treatment over 24 weeks was well tolerated by all rats. Both groups, the control and treatment groups, gained significantly in weight during the observation period without any significant difference between the two groups. Furthermore, there was no significant difference between the groups in organ weight of the heart and kidney (Table 1) and in the plasma concentration of the blood parameter IP. In contrast, Ca plasma levels and ALP plasma concentration increased significantly during long-term AZA treatment. However, the calcium phosphate product was not significantly different between both groups (Table 1). The PTH plasma concentration increased upon AZA treatment for 24 weeks but did not reach statistical significance.

### 3.2. Effect of Azathioprine on Vessel Mineralization after 24 Weeks of Treatment

**Figure 1:**
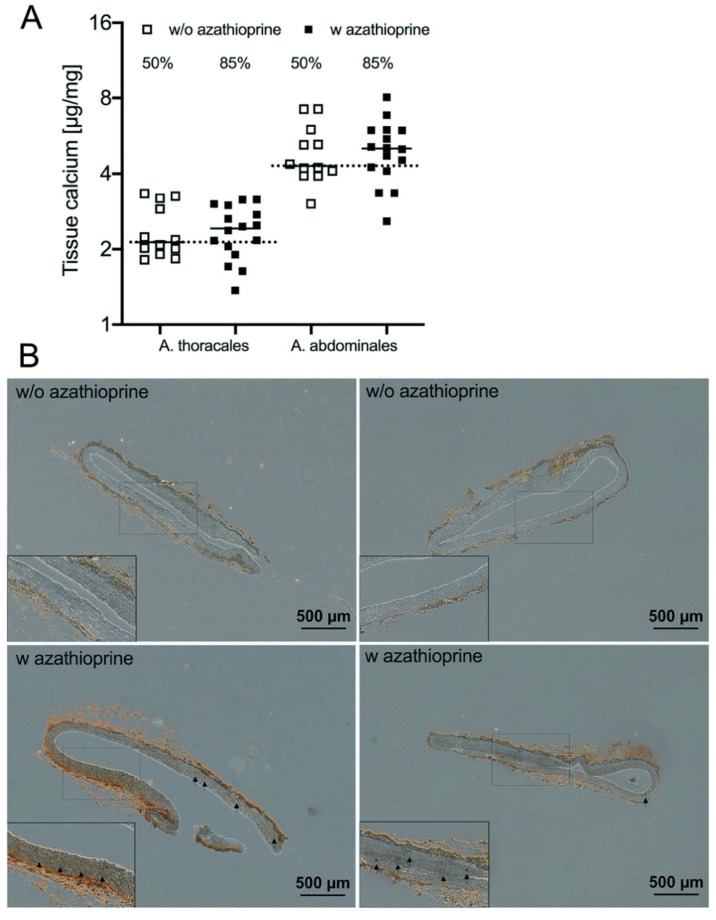
Quantification of mineralization of aortic tissue from rats. (**A**) Distribution and frequency of calcium content in different vessels (median). Each data point represents one animal; the dotted line represents the control median of azathioprine (AZA)-untreated animals; the % represents the number of animals above the control median (w/o AZA: *n* = 12, w AZA: *n* = 16). (**B**) Two representative images per group of von Kossa-stained aortic sections (A. abdominales). Arrow indicates vessel mineralization crystals.

The degree of calcification of the Aorta (A.) thoracales and A. abdominales was determined by measurement of tissue calcium content and via von Kossa staining. The distribution and frequency of the calcium content of investigated vessels are given in Figure 1A. The majority of animals in the AZA-treated group (85%) had elevated tissue calcium in both A. thoracales and A. abdominales. The calcium content was higher in the distal vessel part. The von Kossa-stained histological sections (Figure 1B) of AZA-treated rats demonstrate diffuse calcium deposits localized in the media of the vessel wall, indicating the progression of calcification. The plasma concentrations of the calcification inhibitors Fetuin A and MGP are not different between both groups (Table 2).

### 3.3. Azathioprine Treatment Induces Oxidative Stress and Reduces Antioxidative Capacity in Aortic Tissue

**Figure 2:**
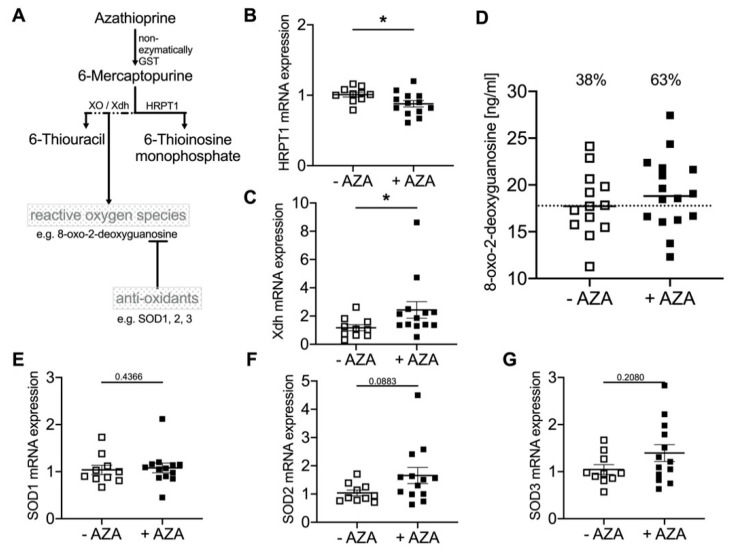
Oxidative and antioxidative capacity upon azathioprine (AZA) treatment. (**A**) Schema of AZA metabolism. (**B**,**C**,**E**–**G**) mRNA expression in aortic tissue from rats (w/o AZA: *n* = 10, w AZA: *n* = 13), Mean ± SEM. * *p* < 0.05 vs. control. (**D**) Reactive oxygen species (ROS) plasma level via detection of 8-oxo-2-deoxyguanosine. Each data point represents an animal (median, w/o AZA: *n* = 13, w AZA: *n* = 16); the dotted line represents the control median of AZA-untreated animals; the % represents the number of animals above the control median.

A short schema of the AZA metabolism is given in Figure 2A. The prodrug AZA is cleaved non-enzymatically or by glutathione S-transferases (GST) to 6-MP, which is then further metabolized enzymatically by xanthine oxidase (XO) and hypoxanthine-guanine phosphoribosyl transferase (HRPT1) to 6-thiouracil or 6-thioionosine monophosphate. After AZA treatment, the HRPT1 mRNA in aortic tissue is significantly decreased (Figure 2B), while XO is significantly increased after AZA treatment (Figure 2C).

During AZA catabolism via XO, reactive oxygen species (ROS) are generated. Therefore, 8-oxo-2-desoxyguanosine plasma levels as a ROS marker were measured. The distribution and frequency of the ROS increase upon AZA treatment are shown in Figure 2D. To investigate the antioxidative capacity, SOD1, 2 and 3 mRNA expression was measured. SOD1 and SOD3 mRNA expression was not found to be different in the AZA-treated group compared to the untreated controls. For SOD2, the mRNA expression slightly increased upon AZA treatment; however, it did not reach statistical significance (Figure 2E–G).

### 3.4. Azathioprine Treatment Induces SASP: Cytokine Plasma Level

**Figure 3:**
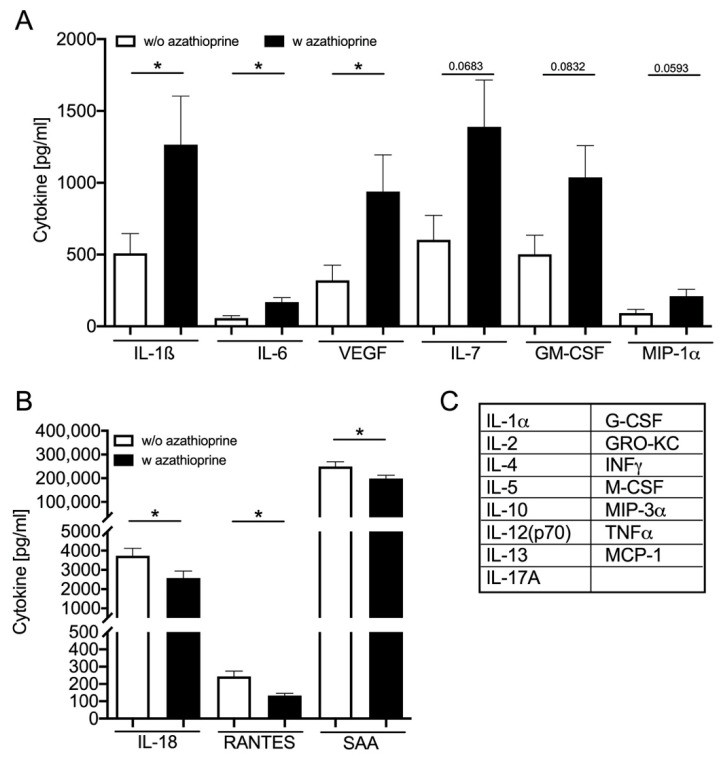
Cytokine plasma concentration in rats. (**A**) Cytokines that increase in plasma upon azathioprine (AZA) treatment and (**B**) that decrease compared to the untreated animals. Mean ± SEM, * *p* < 0.05. (**C**) Further measured cytokines without a significant difference between AZA-untreated and -treated groups.

Secretion of proinflammatory cytokines is one sign of the SASP of cells [19]. Therefore, the plasma levels of different cytokines were measured as systemic markers of inflammation. Out of 23 investigated cytokines, plasma concentrations of six are increased (Figure 3A) and three are decreased (Figure 3B) upon AZA treatment compared to the control group. In the treatment group, the plasma concentration of IL-1ß, IL-6 and vascular endothelial growth factor (VEGF) significantly increased, whereas the concentration of IL-7, GM-CSF and MIP-1α only tends to be increased. Interestingly, the plasma concentration of IL-18, RANTES and serum amyloid A (SAA) significantly decreased in the treatment group.

### 3.5. Azathioprine Treatment Induces SASP and Mineralization in Aortic Tissue

**Figure 4:**
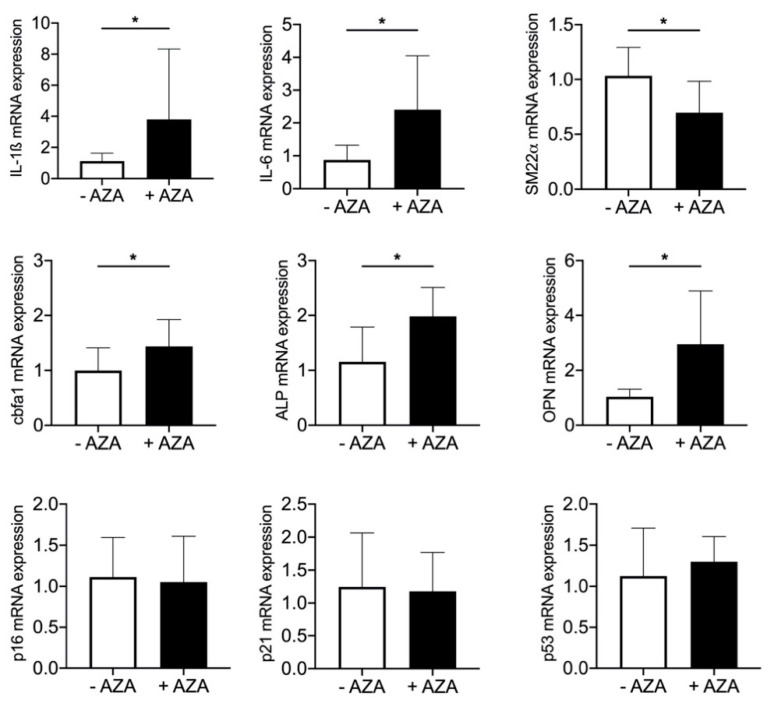
Expression of senescence-associated phenotype markers in aortic tissue from rats upon azathioprine treatment. mRNA expression in aortic tissue of azathioprine (AZA)-treated rats vs. control animals. Mean ± SEM, * *p* < 0.05.

Cells change their expression profile during SASP response [13]. Therefore, the mRNA expression of osteoblastic, inflammatory and senescence markers in aortic tissue was determined. In the treatment group, the expression of the osteoblastic marker protein core-binding factor α1 (Cbfa1), the tissue non-specific ALP and osteopontin (OPN) significantly increased, whereas the expression of the VSMC marker protein SM22α significantly decreased compared to the control group. In addition to the increased systemic expression of the proinflammatory cytokines IL-1ß and IL-6, their mRNA expression in aortic tissue was significantly upregulated after 24 weeks of AZA treatment. The expression of cell cycle proteins p16, p21 and p53 was not altered (Figure 4).

### 3.6. SASP Induction Is NLRP3-Dependent

**Figure 5:**
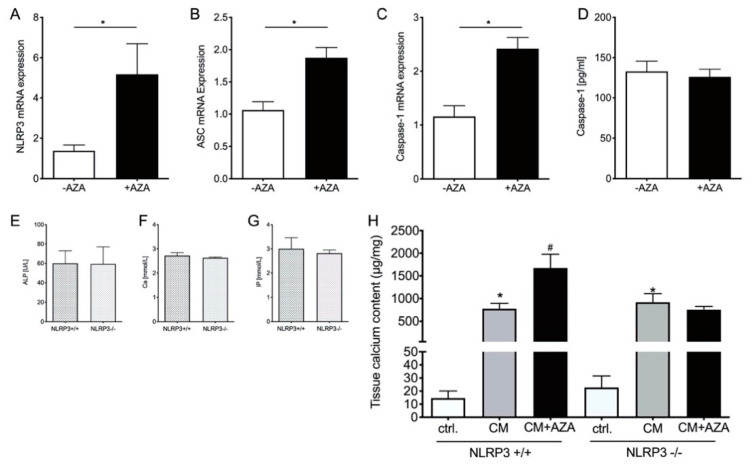
NLRP3-dependent induction of senescence-associated phenotype markers. (**A**–**C**) mRNA expression in aortic tissue from rats. (**D**) Caspase-1 plasma concentration in azathioprine (AZA)-treated and -untreated rats. (**E**–**G**) Plasma concentration of alkaline phosphatase (ALP), inorganic phosphate (IP) and calcium (Ca) in NLRP3^+/+^ and NLRP3^−/−^ mice. (**H**) Aortic tissue rings of NLRP3^+/+^ and NLRP3^−/−^ mice were treated for 14 days ex vivo using control and calcification media in the presence or absence of AZA (0.1 mmol/L) and tissue calcium contents were quantified. Arrows indicating calcification foci. (**E**–**H**) *n* = 6. (**A**–**H**) Mean ± SEM, * *p* < 0.05 vs. control, # *p* < 0.05 vs. CM.

There is evidence that the NLRP3 inflammasome complex is involved in mVC [15]. Therefore, we tested the mRNA expression of ASC, NLRP3 and Caspase-1, in the aortic tissue of our model. In the treatment group, the expression of NLRP3, ASC and Caspase-1 is significantly increased in the aortic tissue (Figure 5A–C). The systemic plasma concentration of Caspase-1 is not significantly different between treated and untreated rats (Figure 5D). To further investigate the pathway via NLRP3 in the calcification process, aortic rings from the A. thoracales of NLRP3^−/−^ mice and respective NLRP3^+/+^ control animals were stimulated ex vivo for 14 days with calcification medium alone and with calcification medium supplemented with AZA. The plasma levels of ALP, IP and Ca do not significantly differ between knockout and control animals. However, in aortic rings of NLRP3^+/+^ mice, calcification media significantly induce tissue calcium content, and co-stimulation with AZA further increases tissue calcium content in these animals. In NLRP3^−/−^ mice, the induction effect of AZA is significantly reduced (Figure 5H).

## 4. Discussion

**Figure 6:**
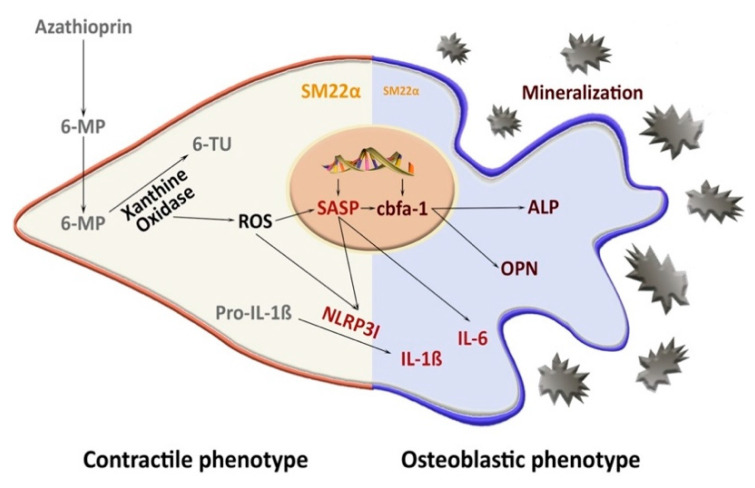
Proposed mechanism of azathioprine-induced vessel mineralization. 6-MP: 6-mercaptopurine, 6-TU: 6-thiouracil, ROS: reactive oxygen species, IL-ß: interleukin 1-ß, IL-6: interleukin 6, SASP: senescence-associated secretory phenotype, NLRP3I: NLRP3 inflammasome, SM22α: smooth muscle protein 22 α, cbfa1: core-binding factor alpha-1, OPN: osteopontin, ALP: alkaline phosphatase.

In the current study, we examined the in vivo long-term effect of AZA treatment on mVC in rats. In line with our in vitro data [10], we show that AZA treatment over 24 weeks induces vessel calcification and proinflammatory SASP activation in the aortic tissue. The effect is, at least in part, driven by the NLRP3 inflammasome (Figure 6).

AZA is a potent immunosuppressive drug that it is still routinely used as a treatment of several auto-immune diseases [7,8,9]. Evidence exists that AZA treatment increases the cardiovascular risk of patients [8,9]. Arteriosclerosis and subsequent stiffening of the vessel wall are some of the risk factors for cardiovascular events, not only in patients with CKD or after organ transplantation [2,3].

In a recent in vitro study, we already observed an induction of VSMC calcification upon 6-MP treatment [10]. 6-MP is the active metabolite of AZA which is cleaved non-enzymatically or via GST [8,20]. In line with the in vitro data, the present study shows an increased mVC of the aortic tissue in the treatment group. However, even though the calcification was induced by AZA treatment, the effect was less robust than expected from the in vitro data. The animals in the current study were treated with 10 mg/kg body weight for 24 weeks. 

For other animal studies, the drug dose of AZA or its active metabolite 6-MP differs between 2 and 60 mg/kg body weight depending on the research question and model [21,22,23,24,25,26,27,28]. The drug dose for humans is usually 1–4 mg/kg body weight and depends on the individual indication. As the dose in animals is mostly higher than in humans, the application time in the animal experiment is relatively short compared to the treatment period of often decades in humans. In line with previous studies for different treatment periods, we found no significant difference in rat body mass upon AZA treatment [24,29].

In contrast to the in vitro situation, where only one cell type is typically investigated in an artificial environment, the in vivo situation is influenced by multiple systemic factors [30] that might contribute to differences observed in vitro and in vivo. The mechanisms in mVC are multifactorial and in vivo triggered by an imbalance between mineralization inducers and inhibitors [11]. First, we looked at blood parameters typically associated with mVC. Plasma concentrations of IP, PTH and the calcium phosphate product do not significantly differ between the treatment and control groups in our rat model. Under pathophysiological conditions, calcium and phosphate concentrations typically exceed their solubility. Endogenous inhibitors such as Fetuin-A and MGP are required to prevent ectopic precipitation of calcium phosphate complexes. Fetuin-A has potent inhibitory effects on calcification by protecting crystal growth and deposition [31]. MGP is highly expressed in calcified VSMCs, acts via a vitamin K-dependent pathway and has a high binding affinity to calcium ions [32]. However, plasma concentrations of Fetuin-A and MPG are not significantly different between the treatment and control groups and therefore cannot explain the observed effects.

As shown in vitro, the induction of calcification upon 6-MP was, at least partially, dependent on ROS generation via XO [10]. Therefore, we investigated the mRNA gene expression of HPRT-1 and XO, the main catabolic enzymes of 6-MP, in the aortic tissue. The expression of HPRT-1 was reduced, whereas the expression of XO was significantly enhanced. The increased plasma level of 8-oxodehydrogenase supports the finding of an ROS-dependent effect of AZA treatment via XO, which is in accordance with our former in vitro results [10]. In vessels from children on dialysis [13], 8-oxodehydrogenase could also be detected in calcified medium-sized muscular arteries, which shows the relevance of ROS for the induction of mVC as discussed elsewhere [33]. In our rat model, the expression of SOD1, 2 and 3 in aortic tissue as a measure of antioxidant capacity was not significantly altered between the groups.

The enhanced production of inflammatory cytokines/chemokines, such as IL-1, IL-6 and VEGF, is associated with the prevalence of mVC and age-related diseases [34,35,36,37]. Therefore, we first measured the systemic plasma levels of 23 potentially relevant analytes from the class of cytokines/chemokines and growth factors with potentially proinflammatory properties in our rat model. Indeed, the known SASP cytokines IL-1ß, IL-6 and VEGF are significantly increased upon AZA treatment. The activation of those SASP cytokines is in line with further research that found an induction of “paracrine senescence” in cell culture as well as mouse models [38]. Furthermore, the plasma level of IL-7, GM-CSF and MIP-1α tends to be increased upon AZA treatment for 24 weeks. IL-7 has been shown to be upregulated in some age-related diseases [34]. Although the main sources of origin of the cytokines are different, cells of the blood vessels such as macrophages, T cells, monocytes and platelets, as well as endothelial cells and VSMC, could also produce cytokines [39].

Beside upregulation of inflammatory cytokines, the plasma levels of IL-18, RANTES and SAA are significantly decreased in AZA-treated animals compared to their respective controls. SAA acts as a proinflammatory cytokine on VSMCs via activation of, among others, toll-like receptor (TLR) 2 and 4 to activate monocyte chemoattractant protein-1 production (MCP-1) [40]. MCP-1 and RANTES have chemoattractive properties on macrophages in the vessel wall [21]. Pols et al. found an inhibition of MCP-1 upon 6-MP treatment, whereas no significant effects could be shown on RANTES production [21]. The atheroprotective properties of 6-MP were observed in a mouse model of hypercholesterolemic apoE animals, whereby the inhibitory action was mainly triggered by a reduction in monocyte activation [21]. Beside the detection of systemic SASP markers, we investigated the SASP activation directly in aortic tissue. Here again, the mRNA expression of IL-1ß and IL-6 is significantly increased upon AZA treatment. IL-6 is known as an inducer of mineralization in VSMCs [16,41,42]. In part, IL-6 influences MPG’s inhibitory action [43]. In addition, IL-6 induces bone morphogenetic protein (BMP)-2, ALP and OPN expression in VSMCs in vitro [42]. The neutralization of IL-6 reduced the osteogenic mRNA expression in an in vitro model [44]. For IL-1ß, a calcification induction in VSMCs was also shown [15]. Further, typical osteogenic markers such as the transcription factor Cbfa1 and its downstream genes ALP and OPN significantly increase in the aortic tissue of AZA-treated animals. Cbfa1, also known as Runx2, is not only a regulatory transcription factor of osteoblastic differentiation but also an initial osteoblastic differentiation factor of VSMCs that is required for medial calcification in mice [45,46]. OPN provides strong affinity for hydroxyapatite, due to its negatively charged phosphoserines, and therefore acts as an inhibitor of mVC by preventing crystal growth [47]. In healthy arteries, OPN is not detectable but was found increased in calcified tissue [48]. The inhibitory effect of OPN on mVC has been shown in vitro and in vivo [49,50,51]. As all of these proteins are upregulated in aortic tissue upon AZA treatment, this underlines the harmful effect on vessel physiology. Although the inductions of senescence markers such as p16, p21 and p53 are also described as SASP components, we found no significant induction of those in our model upon AZA treatment. The missing induction of the investigated senescence markers p21, p16 and p53 in the aortic tissue might be one explanation for the lesser calcification induction compared to our in vitro model [10]. Here, AZA induces p16, p21 and p53 (unpublished data). Induction of VSMC calcification by hyperphosphatemia also induces p21 [52]. However, IL-6 production itself is a sign of senescent VSMCs [53]. Furthermore, IL-1ß induces senescence of VSMCs and promotes osteogenic differentiation [35]. Several pieces of evidence exist of a vicious cycle of calcification and senescence in VMSCs; however, whether this is a conjoint or consecutive pathway is not clear up to now. Microarray analysis of senescent VSMCs reveals differential regulation of calcifying markers such as MGP, BMP2 and OPG as well as inflammation markers such as IL-1ß and tissue remodeling markers such as VEGF [36]. However, one has to keep in mind that some of the experimental settings investigate bulk cells and single-cell-based methods seem to be necessary to answer that question.

To further examine the intracellular signaling pathway of SASP induction in the aortic tissue, we investigated the NLRP3 inflammasome, which is believed to play a role in the mineralization process and contribute to age-related disease [15,34,54]. NLRP3, ASC and Caspase-1 are upregulated in calcified VSMCs and lead to a subsequent IL-ß production [15]. The inhibition of the NLRP3 prevents calcification in vitro [15]. In the aortic tissue of AZA-treated rats, we found a significant increase in NLRP3, ASC and Caspase-1 mRNA expression, which is in line with the detected aortic IL-1ß expression. However, the systemic Caspase-1 plasma levels were not found to be significantly induced upon AZA treatment. To further verify the critical role of NLRP3 activation in our model, we studied aortic tissue in an ex vivo setting for 14 days upon AZA incubation. Our results confirm that NLRP3 inflammasome activation may be, at least, one crucial signaling pathway in AZA-induced mVC.

Limitations of the study: The current study investigated vessel alterations such as media calcification upon AZA treatment. We wanted to investigate the effect of AZA on adult rats and therefore started the six-month treatment at the age of 14 weeks. Although only slight mineralization foci were found in the aortic tissue, a more extended treatment period was not possible due to the 3R (Reduce, Replace, Refine) thought of Russel and Burch [55] due to the increased tumor risk in older rats [56]. The current study did not investigate vessel stiffness parameters upon treatment such as pulse wave velocity, pulse pressure and systolic/diastolic blood pressure. However, the mRNA expression profile in the aortic tissue shows changes in the expression pattern of typical marker proteins for stiffened, mineralized vessels. Here, bone remodeling was not investigated. However, from other studies, it is known that AZA treatment in rats induces bone remodeling disorders by inhibiting bone formation and bone mineralization [29].

## 5. Conclusions

In conclusion, the current study confirmed the possible harmful effect of AZA treatment on vessel structure that was previously found in vitro using rat VSMCs [10]. Beside mineralization foci in the media of the aortic vessel wall, several changes in the SASP profile could be detected in the aortic tissue. Even though the effect seems not to be as strong as expected from our in vitro results [10], one has to keep in mind that humans are treated over years with AZA, whereas in the current model, only a smaller period could be investigated. In addition, AZA-treated patients mostly suffer from several comorbidities that could further reduce the antioxidative capacity of the organism and therefore pronounce the AZA-induced oxidative stress effect on VSMCs. However, the hypothesis has to be confirmed in human studies.

## Figures and Tables

**Table 1:** Body weight, blood parameters and organ weight of rats. Heart and kidney weights are given as wet tissue weight normalized to animal weight. Mean ± SEM.

Group	Rat N	Body Weight [g] Initial vs. Final	Heart Weight [mg/g]	Kidney Weight [mg/g]	ALP [U/L]	IP [mmol/L]	Ca [mmol/L]	Ca x IP	PTH [pg/mL]
**Control**	13	332.1 ± 6.2 **504.2 ± 10.5 ^#^**	2.54 ± 0.06	5.09 ± 0.10	148.50 ± 18.49	2.03 ± 0.12	2.41 ± 0.07	4.9 ± 0.34	888.0 ± 155.4
**AZA**	16	344.9 ± 5.0 **511.4 ± 11.9 ^#^**	2.60 ± 0.09	5.21 ± 0.13	204.30 ^1^ ± 23.54 *	2.10 ± 0.07	2.63 ± 0.08 *	5.5 ± 0.25	1.347.0 ± 387.7

AZA: azathioprine, ALP: alkaline phosphatase, IP: inorganic phosphate, Ca: calcium, PTH: parathyroid hormone. ^1^ Animal number 14, ^#^ *p* < 0.05 initial vs. final of the same group, * *p* < 0.05 control vs. AZA.

**Table 2:** Plasma concentration of calcification inhibitors. Mean ± SEM.

Group	Rat Number	Fetuin-A [ng/mL]	MGP [ng/mL]
**Control**	13	89.22 ± 0.28	92.25 ± 4.17
**AZA**	16	89.21 ± 0.17	84.42 ± 2.81

AZA: azathioprine, MGP: matrix Gla protein.

## Supplementary Materials

Supplementary materials are available online at https://www.mdpi.com/2227-9059/9/3/327/s1.
