# Supplementary material for: Long-Term Treatment of Azathioprine in Rats Induces Vessel Mineralization
Source: Biomedicines. 2021 Mar 23;9(3):327. doi: 10.3390/biomedicines9030327 (PMC8004774; doi:10.3390/biomedicines9030327)
Supplement: Supplementary file 1 [file biomedicines-09-00327-s001.pdf]

Supplementary Materials

Suppl. Table S1. Oligonucleotide sequences rat

**Gene**	**Fwd 5´-3´**<br>**Rev 5´-3´**	**Company**	**Reference**
HRPT-1	GAC TTT GCT TTC CTT GGT CA<br>AGT CAA GGG CAT ATC CAA CA	Biozol, Eching	Order Nr.<br>RHK-1
Xdh	TGG ACA AGT AGA GGG GGC AT<br>ACA CAG GCG TTT CGG ATC TT	Tibmolbiol, Berlin	Primer<br>Blast
SOD1	TTT TGC TCT CCC AGG TTC CG<br>CCC ATG CTC GCC TTC AGT TA	Tibmolbiol, Berlin	Primer<br>Blast
SOD2	CAC CGA GGA GAA GTA CCA CG<br>TGG GTT CTC CAC CAC CCT TA	Tibmolbiol, Berlin	Primer<br>Blast
SOD3	GAG AGC TTG TCA GGT GTG GA<br>GTC AAG CCG GTC TGC TAA GT	Tibmolbiol, Berlin	Primer<br>Blast
Cbfa1	GCC GGG AAT GAT GAG AAC TA<br>GGA CCG TCC ACT GTC ACT TT	Tibmolbiol, Berlin	Primer<br>Blast
ALP	TCC GTG GGT CGG ATT CCT<br>GCC GGC CCA AGA GAG AAA	Tibmolbiol, Berlin	[48]
OPN	TGG TTT GCC TTT GCC TGT TC<br>TCT CCT CTG AGC TGC CAA ACT C	Tibmolbiol, Berlin	Primer<br>Blast
SM22α	AGA GGG GCC TCA CAG GCT GG<br>ACA GCT GGG AAC AGG GGC CA	Tibmolbiol, Berlin	Primer<br>Blast

p53	AGC TCC AGT TCA TTG GGA CTT<br>CAG TTA TCC AGT CTT CAG GGG A	Tibmolbiol, Berlin	Primer Blast
NLRP3	TCT CTG CAT GCC GTA TCT GG<br>ACG GCG TTA GCA GAA ATC CA	Tibmolbiol, Berlin	Primer Blast
Caspase-1	GGA GCT TCA GTC AGG TCC ATC<br>CTT GAG GGA ACC ACT CGG TC	Tibmolbiol, Berlin	Primer Blast
ASC	TTA TGG AAG AGT CTG GAG CTG TG<br>GCA ATG AGT GCT TGC CTG TG	Tibmolbiol, Berlin	Primer Blast
p16 (Cdkn2a)	CAG ATT CGA ACT GCG AGG AC<br>CCC AGC GGA GGA GAG TAG AT	Biomol, Hamburg	Order Nr. VRPS-1014
p21 (Cdkn1a)	TGG TCC TTT CCC AGT ATT GA<br>CAC GTG GGA GGT TTA CAA TC	Biomol, Hamburg	Order Nr. VRPS-1011
IL-1ß	AGA GTG TGG ATC CCA AAC AA<br>AGT CAA CTA TGT CCC GAC CA	Biomol, Hamburg	Order Nr. VRPS-2929
IL-6	CTT CCT ACC CCA ACT TCC AA<br>ACC ACA GTG AGG AAT GTC CA	Biomol, Hamburg	Order Nr. VRPS-2952
GAPD	AGA CAG CCG CAT CTT CTT GT<br>CTT GCC GTG GGT AGA GTC AT	Biozol, Eching	Order Nr. RHK-1
Ppia	CTG GTG GCA AGT CCA TCT AC<br>CCC GCA AGT CAA AGA AAT TA	Biozol, Eching	Order Nr. RHK-1
Rpl13A	GTG AGG GCA TCA ACA TTT CT<br>CAT CCG CTT TTT CTT GTC AT	Biozol, Eching	Order Nr. RHK-1
